# 

**Published:** 1915-08-21

**Authors:** 


### CONTENTS.

| | PAGE |
|---|---|
| **Hindrances to Medical Recruiting** ... ... | 423 |
| **Antiseptics and the War** ... ... ... ... ... | 424 |
| **Hospital and Institutional News** ... ... ... | 425 |
| Our Naval Medical Heroes—Surgeon Langford's D.S.O.—The Star and Garter as a Home of Rest—A White Elephant or a Gift?—The Wandsworth Union's Institutions and the Progress Made—Reductions in an Infirmary Dietary—A Panel Doctor Ordered out of Court—Medical Treatment for Soldiers on Furlough—Insurance against Air Raids at Worcester—The Needs of the French Red Cross—Asylum Agitation in Australia—An R.A.M.C. Officer's Death—Shortage of Doctors in Australia—German Firms with English Pretensions—Water Sterilisers for Belgian Soldiers—Hospital War Work at Melbourne and Sydney—Thefts from the London Hospitals—The British Hospitals Association—The Training of Medical Women—The Anglo-Russian Hospital or Russian Flag Day Fund?—The Truth about Overlapping—The Boom of the Bluebottle—The Resources of Science against the Fly—This Week's Drug Market. | |
| **The Army Chaplain and the R.A.M.C.** ... | 431 |
| The Change of System on its Trial. | |
| **Air-Raids and Bombardment of Institutions** | 432 |
| The Government Insurance Scheme. | |
| **A Medical Causerie** ... ... ... ... ... | 433 |
| **Round the World** ... ... ... ... ... | 434 |
| Fellow-Workers and the Roll of Honour. | |
| **Poor-Law Medical Appointments** ... ... | 435 |
| The Local Government Board and Guardians' Contracts. | |
| **Legal Notes for Institutional Workers** ... | 436 |
| Institutional Friction—Heavy Damages for a Doctor. | |
| **The Editor's Letter-Box** ... ... ... ... | 437 |
| "Religio Medici Modereni." | |
| Heating of Leicester Infirmary. | |
| Female Nurses in Asylum Male Wards. | |
| **The New Physiological Buildings at Cardiff** ... | 438 |
| The Question of a Treasury Grant. | |
| **The War's Effect on an East Coast Hospital** | 439 |
| New Work and its Problems at Ipswich. | |
| **Buildings Contemplated and in Progress** ... | 440 |
| **Tenders** ... ... ... ... ... ... ... | 440 |
| **Endowed Beds with a History** ... ... ... | 441 |
| I.—In the London Hospitals. | |
| **Gifts and Bequests** ... ... ... ... ... | 442 |
| **Latest Installations in Hospital Equipment** ... | 442 |
| **The Book Market** ... ... ... ... ... ... | 442 |
| **The Institutional Worker** ... ... (Suppl.) | 1 |
| Economy in Institutions: The Disposal of Broken and Disused Material. | |
| Question for August. | |
| Enquiries and Answers. | |
| Employment and Training. | |
| War Matters. | |
| Miscellaneous. | |

